# Hawthorn Polysaccharides Relieve High-Fat Blood Disease in Mice by Improving Intestinal Flora and Promoting Lipid Metabolism

**DOI:** 10.3390/foods15091525

**Published:** 2026-04-28

**Authors:** Jingxuan Ke, Xinyu Li, Xiaoyu Yin, Yabin Wang, Xin Wang, Qingshan Shen, Yanli Ma

**Affiliations:** 1 Zhang Zhongjing College of Health Preservation and Food Science, Nanyang Institute of Technology, Nanyang 473004, China; jingxke@163.com (J.K.); lixinyu20005@outlook.com (X.L.); 18837738196@163.com (X.Y.); devid7758521@outlook.com (Y.W.); shenqs973@163.com (Q.S.); 2 College of Food Science and Technology, Hebei Agricultural University, Baoding 071000, China; wx110100@163.com

**Keywords:** hawthorn polysaccharides, alleviate hyperlipidemia, high-fat diet, lipid metabolism

## Abstract

In this research, the anti-hyperlipidemic effects of hawthorn polysaccharides in experimental mice fed a high-fat diet were thoroughly investigated. The findings indicated that the body and organ weights of the high-fat group (HC) mice increased significantly, fat accumulation was evident, and serum indicators showed elevated lipid levels. After 8 weeks of the intragastric administration of hawthorn polysaccharides, the data showed that the body weight of the hawthorn polysaccharide (HA) group was notably lower than that of the HC group and close to that of the NC group. In addition, the hawthorn polysaccharide intervention improved the symptoms of the mice. In particular, the hawthorn polysaccharide intervention significantly increased HDL-C levels and decreased LDL-C levels in the HA group mice. Furthermore, gut microbiota analysis demonstrated that a high-fat diet altered its structure. The intervention with hawthorn polysaccharides modulated the intestinal flora structure, lowered the F/B ratio, and increased the abundance of beneficial bacterial strains associated with lower blood lipid levels.

## 1. Introduction

With the improvements in people’s living standards, high-calorie diets and a lack of exercise have led to hyperlipidemia becoming a prevalent metabolic disease [1,2]. A disorder of lipid metabolism causes hyperlipidemia [3,4,5]. Statins are currently the preferred drugs in clinical treatment [6], but they can cause specific side effects and discomfort. Therefore, identifying safe, active food components that can improve lipid metabolism has become a key research focus. Numerous studies have shown that polysaccharides [7,8], dietary fibers [9,10], and flavonoids [11] in plant-based foods exhibit lipid-lowering effects. Plant polysaccharides, with their mild, safe, and lipid metabolism-regulating properties, have become a new research trend [12].

Modern pharmacological investigations have demonstrated that polysaccharides can regulate oxidant stress and enhance lipid metabolism [13]. Animal investigations have shown that polysaccharides can help alleviate morbid obesity and steroidogenic hyperlipidemia caused by a high-fat diet. The research conducted by Rjeibi et al. [14] demonstrated that the polysaccharides (NRFPs) found in the fruits of Nitraria retusa can treat hyperlipidemia [14]. Additionally, Nie et al. (2017) showed that rice husk polysaccharides noticeably lowered lipids in mice fed a high-fat diet by regulating live lipid metabolism [7]. Another study also demonstrated that flaxseed polysaccharides effectively alleviate metabolic syndrome by modulating the expression of genes related to fat metabolism, modulating the intestinal microbiota, and restoring the synthesis of volatile fatty acids [15].

Research on the lipid-lowering effects of natural plant polysaccharides has attracted considerable attention. However, the mechanisms vary. Numerous animal experiments have demonstrated that regulating the structure of intestinal flora is a primary theory for the way that plant polysaccharides exert their lipid-lowering effects. The long-time intake of high-fat diets increases the burden on the intestines, disrupts the regularity of the intestinal flora, damages the intestinal mucosal barrier, promotes the infiltration of inflammatory cells and the activity of inflammatory factors in tissues, and ultimately leads to lipid metabolic disorders [16].

Plant polysaccharides can reduce the intestinal burden associated with a high-fat diet by accommodating the constituents and functions of the intestinal flora [17,18], thereby restoring intestinal microorganisms to a normal state and alleviating gut microbiota imbalance. Additionally, plant polysaccharides could increase the quantity of beneficial intestinal microorganisms, thereby helping prevent obesity and related diseases [19].

Li et al. (2023) found that, when the diets of high-fat diet mice were supplemented with citrus polysaccharides (PCRCPs), the PCRCPs could restrict obesity-related bacteria and, at the same time, accelerate the reproduction of obesity-resistant bacteria [20]. The study by Ke et al. [8] suggested that polysaccharides of *Platycodon grandiflorus* effectively reduce weight gain and extreme fat accumulation generated by a high-energy diet. In conclusion, the lipid-lowering effect of PS was achieved by altering the structure and metabolic activity of the intestinal microbial community [8]. Evidence from the above literature has shown that plant polysaccharides can alleviate high blood lipid levels and obesity caused by a high-energy diet by regulating the composition of the intestinal microbial community. Therefore, further scientific studies on the mechanism by which the composition of the gut microbial community is modulated by plant polysaccharides are needed to enable targeted dietary interventions for the gut microbiota, to prevent or alleviate hyperlipidemia.

Hawthorn (Crataegus) is the dried and mature fruit of a plant from the genus Crataegus [21]. Modern research has confirmed that it contains a large number of miscellaneous functional components, incorporating polysaccharides, phenolic acids, and organic acids [22]. Hawthorn polysaccharides are an essential type of water-soluble biological macromolecule in hawthorn. Hawthorn polysaccharides exhibit good biological safety and have demonstrated various pharmacological activities, including antioxidant activity [23], the regulation of sugar [24] and lipid metabolism [25,26], and the protection of the gastrointestinal mucosa [22], in both simulation experiments and animal experiments [22]. They have extensive applications in research on healthy food and natural medicine. Currently, most research on hawthorn polysaccharides focuses on the in vitro verification of specific activities. The in vivo mechanisms underlying certain activities (such as the regulation of the gut microbiota and the reduction in blood lipids) still need to be further explored. Exploring these issues could provide key theoretical support for the external application of hawthorn polysaccharides.

In this study, the effects of a hawthorn polysaccharide diet intervention on hyperlipidemia in mice fed a high-fat diet and the mechanisms underlying such were thoroughly investigated. First, mice on a high-fat diet underwent an 8-week intervention with a hawthorn polysaccharide diet. Then, changes in body weight and fat accumulation in mice following the hawthorn polysaccharide intervention were examined. Next, the effects of hawthorn polysaccharides on the serum indicators and liver antioxidant indicators of mice fed a high-fat diet were analyzed. Finally, by analyzing the gut microbiota’s structure, the mechanism of action of hawthorn polysaccharides in treating hyperlipidemia induced by a high-fat diet was elucidated.

## 2. Materials and Methods

### 2.1. Materials

The hawthorn polysaccharides applied in this experiment were purchased from Xi’an Kepin Biotechnology Co., Ltd. (Xi’an, China). The extraction method for hawthorn polysaccharides is water extraction followed by alcohol precipitation. The brief extraction process is as follows: Soak the hawthorn residue in hot water (100 °C) 8 times, and extract for one hour. Next, add 5 times the volume of 85% ethanol solution to the supernatant. Then, leave the mixture to stand for 24 h for precipitation. Collect the precipitate and dry it to acquire the hawthorn polysaccharides.

### 2.2. Characterization of Hawthorn Polysaccharides’ Properties

#### 2.2.1. Purity of Hawthorn Polysaccharides

The purity of the hawthorn polysaccharides was estimated using the phenol–sulfuric acid method [27]. The protein content in the hawthorn polysaccharides was determined by the Coomassie brilliant blue method.

#### 2.2.2. Analysis of Molecular Weight and Composition of Monosaccharides

The molecular weight of the hawthorn polysaccharides was detected using gel chromatography, based on the method described in [28]. The monosaccharide composition of the hawthorn polysaccharides was measured by high-performance ion-exchange chromatography (HPIEC) following the reported methods [28].

### 2.3. Animal Experiment

The animal experiments adhered to the requirements of the ethical guidelines of the Experimental Animal Ethics Committee of Nanyang Institute of Technology ([2024]001). A total of 24 male mice (C57BL/6J, SPF grade), aged 6 weeks and weighing 18 ± 2 g, were obtained from Beijing Vital River Laboratory Animal Technology Co., Ltd. (Beijing, China).

**Figure 1:**
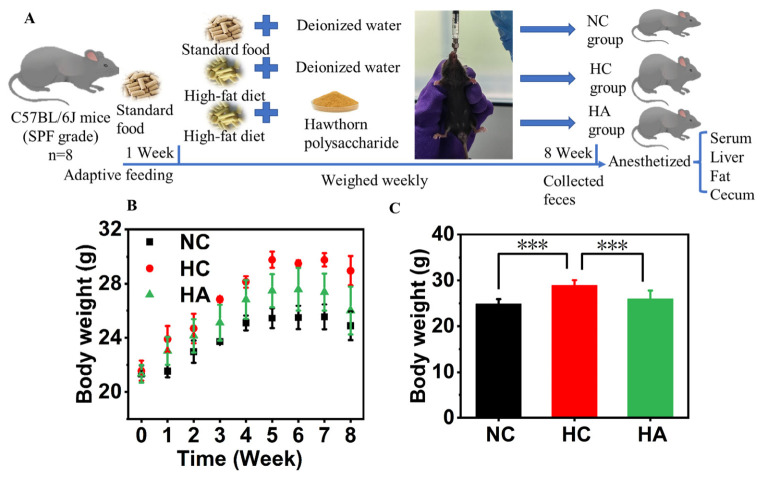
Effects of hawthorn polysaccharide on high-fat-diet mice. (**A**) The design of the intervention to determine the effect of hawthorn polysaccharides on hyperlipidemia in mice fed a high-fat diet. (**B**) The trend of weight gain in mice during the 8-week feeding period. (**C**) The mice’s body weights across the three experimental groups were measured following the 8-week intervention. Values are presented as means ± SEMs, with n = 6 per group; *** *p* < 0.001.

The feeding environment for mice is an SPE environment, characterized by a 12 h day and 12 h night cycle, with the ambient temperature maintained at 22 ± 2 °C and humidity at 55 ± 10% [29]. The mice had free access to food and water throughout the experiment. All the mice underwent a one-week adaptation period and were then separated into three groups at random, with eight animals in each group (Figure 1). Among them, 4 mice were housed in one cage. Data from one or two animals identified as outliers were excluded from the analysis. Normal control (NC group) mice were fed a normal chow diet, whereas the high-fat (HF group) mice received a high-fat diet. The hawthorn polysaccharide diet group (HA group) was also fed a high-fat diet, along with the intragastric administration of hawthorn polysaccharide at 600 mg/kg·BW [23,24].

### 2.4. Determination of Body Weight and Collection of Tissues

During the 8-week feeding experiment, the mice’s weights were measured weekly. On completion of the 8-week intervention, fresh feces were collected from the mice into sterile tubes. Then, the animals were fasted for 12 h without water restriction. Finally, the animals were narcotized with isoflurane. The eyeball was removed to obtain a blood sample, which was centrifuged at 2055 *g* for 15 min to isolate the serum. This was stored at −80 °C before conducting the test. After dissection, the mouse organs (liver, kidney, spleen, heart, perirenal fat, and epididymis fat) were collected and weighed. Then, the contents of the mouse’s cecum were collected. The liver was divided into two parts after weighing; one part was stored at −80 °C, and the other was stored in 4% paraformaldehyde. The kidney weight was calculated as the total weight of both the right and left kidneys. The organ index refers to the ratio of the weight of an organ to the body weight.

### 2.5. Serum Biochemical Indices

Biochemical indices in the blood serum—including total cholesterol (TC), triglycerides (TGs), fasting blood glucose (GLU), aspartate aminotransferase (AST), alanine aminotransferase (ALT), high-density-lipoprotein cholesterol (HDL-C), and low-density-lipoprotein cholesterol (LDL-C)—were determined according to the instructions of the kits (Shandong Biobase Industry Co., Ltd., Jinan, China).

### 2.6. Histological Analysis

The epididymis fat and liver were soaked in paraformaldehyde (4%) and then stuffed. The sections were cut into 4-micron-thick slices and then stained with hematoxylin–eosin (H&E) [15]. Subsequently, they were observed and photographed using an optical microscope (Olympus Corporation, Tokyo, Japan).

### 2.7. Biochemical Indices of Liver Tissue

The antioxidant status in mouse liver tissue was assessed via the detection of the glutathione peroxidase (GSH-PX) and superoxide dismutase (SOD) activities, the malondialdehyde (MDA) level, and the total antioxidant capacity (T-AOC), according to the instructions of the commercial assay kits (Jiangnan Jiancheng Biotechnology Company, Nanjing, China).

### 2.8. Gut Microbiota Analysis

#### 2.8.1. Data Quality Control

Total DNA was isolated from freshly collected mouse feces, followed by PCR amplification targeting the V3–V4 region of the 16S rRNA gene. The primer 341F (CCTAYGGGRBGCASCAG) and primer 806R (GGACTACNNGGGTATCTAAT) were used as the forward and reverse primers, respectively. Based on the Barcode sequence and the PCR amplification primer sequence, the data of each sample were extracted from the off-machine data. After removing the Barcode and primer sequences, FLASH (version 1.2.11, http://ccb.jhu.edu/software/FLASH/, accessed on 7 January 2025) was used to concatenate the reads of each sample, resulting in the concatenated sequences, which were the original Tags data (Raw Tags). Subsequently, the Cutadapt 4.1 software was used to match the reverse primer sequences and cut off the remaining sequences to prevent interference in subsequent analyses. The fastp software (version 0.23.1) was used to perform strict filtering of the raw tags generated by sequencing, resulting in high-quality Tag data (Clean Tags). The Tag sequences were compared with the species annotation databases (Silva database https://www.arb-silva.de/ for 16S/18S, Unite database https://unite.ut.ee/ for ITS, accessed on 7 January 2025) to detect chimeric sequences, which were then removed to obtain the final valid data.

#### 2.8.2. Noise Reduction and Species Annotation of ASVs

For the obtained Effective Tags, noise reduction was performed using the DADA2 module in QIIME2 (version QIME2-202202) or deblur (default using DADA2), thereby obtaining the final ASVs (AmpliconSequence Variants, which are the variations of amplified sequences) and the feature table.

#### 2.8.3. Sample Complexity Analysis

The QIME2 software (version QIME2-202202) was used to calculate the observed_otus, shannonsimpsonchao1goods_coverage, dominance, and pielou_e indices. To assess the complexity of the community composition and compare differences between samples (groups), in QIME2, beta diversity analysis was conducted using weighted and unweighted distances. Principal Component Analysis (PCA) was used to reduce the dimensionality of the original variables using the ade4 package in R and the ggplot2 package (version 4.0.3).

### 2.9. Statistical Analysis

One-way analysis of variance was applied to the data, with Duncan’s test used for subsequent pairwise comparisons. Graphs were created using Origin 2024 software (version 2024, OriginLab, Northampton, MA, USA). The results are presented as mean ± standard deviation (*n* = 6). Statistically significant differences were defined as *p* < 0.05, 0.01, or 0.001. *: *p* < 0.05; **: *p* < 0.01; ***: *p* < 0.001.

## 3. Results

### 3.1. The Basic Characteristics of Hawthorn Polysaccharides

**Figure A1:**
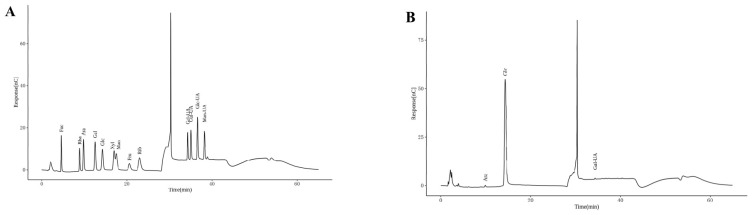
(**A**) Total ion current chromatogram of the standard curve for monosaccharide composition. (**B**) Total ion current chromatogram of the monosaccharide composition of hawthorn polysaccharides.

**Figure A2:**
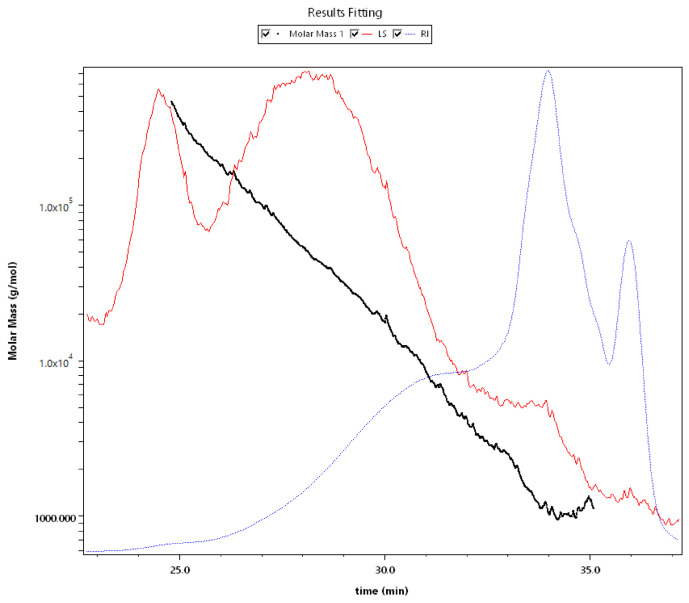
Absolute molecular weight analysis diagram of hawthorn polysaccharides.

The purity of the hawthorn polysaccharides was 81.0%, and the protein content was 16.8% (Figure A1B). The weight-average molecular weight (Mw) and number-average molecular weight (Mn) of the PCP were measured, and the absolute molecular weight analysis diagram is shown in Figure A2. The results indicated that the Mw and Mn were 12.353 kDa and 2.112 kDa, respectively. The monosaccharide composition data showed that hawthorn polysaccharides consisted of Ara (0.89%) and Glc (98.18%), respectively, as shown in Figure A1B. This indicates that the main component of hawthorn polysaccharides may be glucans [30].

### 3.2. Hawthorn Polysaccharides Alleviate Body Weight

The design of the experiment testing the effect of hawthorn polysaccharides on hyperlipidemia in mice fed a high-fat diet is exhibited in Figure 1A. The body weight gain trends for the three experimental mouse groups over the 8-week feeding period are presented in Figure 1B. The HC group showed the fastest weight gain. After adding hawthorn polysaccharides to the diet, the weight gain trend in the HA group was partially mitigated. The weight of the HA group mice was similar to that of the NC group (*p* > 0.05). At the end of the experiment, the HC group weighed 28.97 ± 1.08 g, which was markedly higher than that of the NC group (24.88 ± 1.05 g) (*p* < 0.001), exceeding the NC group’s weight by 16.41%. Therefore, the HC group could be identified as obese model mice [31]. After 8 weeks of hawthorn diet supplementation, there was a remarkable restraining impact on weight gain in mice on a high-calorie diet. As depicted in Figure 1C, the weight of the HA group was 26.02 ± 1.77 g, which was meaningfully lower than that in the HC group (*p* < 0.001); however, the difference was not statistically significant relative to the NC group (*p* > 0.05). The findings demonstrated that hawthorn polysaccharides significantly inhibit obesity caused by a high-fat diet.

### 3.3. The Effects on the Organ and Fat Weights of Mice

**Figure 2:**
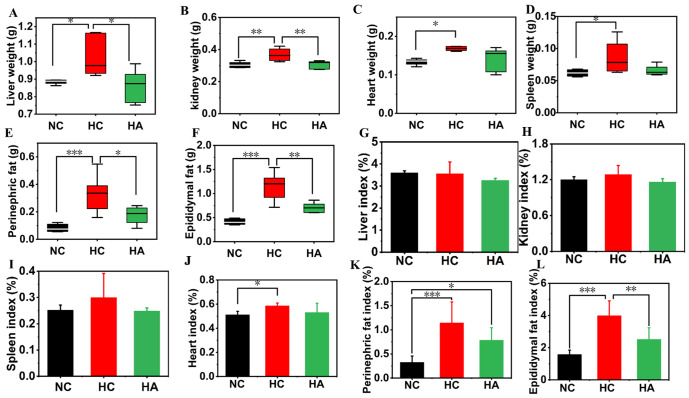
The effects of hawthorn polysaccharide supplementation on organ and fat weight and index in mice fed with a high-fat diet. (**A**) Liver weight; (**B**) kidney weight; (**C**) Heart weight; (**D**) Spleen weight; (**E**) Perinephric fat; (**F**) Epididymal fat; (**G**) Liver index; (**H**) Kidney index; (**I**) spleen index; (**J**) heart index; (**K**) perinephric fat index; (**L**) epididymal fat index. Values are presented as means ± SEMs (n = 6); * *p* < 0.05, ** *p* < 0.01, *** *p* < 0.001.

Compared with a normal diet, the administration of a high-fat diet led to a significant increase in organ and fat weights in mice in the HC group (Figure 2A–D). The addition of hawthorn polysaccharides effectively alleviated this symptom. The weights of the liver, kidney, heart, and spleen of the mice in the HC group were 1.02 ± 0.11 g, 0.37 ± 0.04 g, 0.17 ± 0.01 g, and 0.09 ± 0.03 g, respectively, which were observably higher than those of the NC group (0.89 ± 0.03 g, *p* < 0.05; 0.31 ± 0.02 g, *p* < 0.01; 0.13 ± 0.01 g, *p* < 0.05; 0.06 ± 0.01 g, *p* < 0.05). After hawthorn polysaccharide intervention, this trend was reversed. The weights of the liver, kidney, heart, and spleen of the mice in the HA group decreased to 0.86 ± 0.09 g, 0.31 ± 0.02 g, 0.14 ± 0.03 g, and 0.07 ± 0.01 g, respectively, even reaching the levels of the NC group. The organ index calculation revealed that the heart index of HC group mice was noticeably higher than that of the NC group (*p* < 0.05) (Figure 2G–J). After the supplementation of hawthorn polysaccharides, the heart index decreased, and the heart index in the HA group was close to that of the NC group, without significant differences (*p* > 0.05). Regarding the indices of other organs, non-significant variations were observed across the three groups (*p* > 0.05). On the other hand, a high-fat diet significantly increased the perirenal fat (0.33 ± 0.14 g, *p* < 0.001) and epididymal fat weight (3.99 ± 0.92 g, *p* < 0.001) of the HC group mice, in contrast to the normal diet mice (0.08 ± 0.03 g and 1.58 ± 0.27 g, respectively) (Figure 2E,F). The hawthorn polysaccharide intervention reduced the fat accumulation in mice, and the perirenal fat weight (0.18 ± 0.06 g, *p* < 0.05) and epididymal fat weight (2.52 ± 0.72 g, *p* < 0.01) of the HA group mice were dramatically inferior to those of the HC group, approaching the value of the NC group. These findings were similar to those of Lee et al. (2024) [32]. Similarly, a high-fat diet significantly increased the perirenal and epididymal fat indices (Figure 2K,L, *p* < 0.001). The intervention with hawthorn polysaccharides reversed this trend. It is demonstrated that the hawthorn polysaccharides could reduce the visceral overweight in the high-caloric-feeding mice.

### 3.4. The Impact on Serum Lipid Levels of Mice

**Figure 3:**
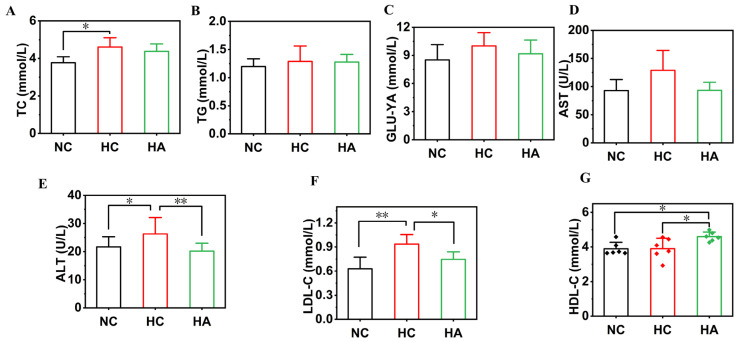
The effects of hawthorn polysaccharide supplementation on the lipid levels of mice on a high-fat diet. (**A**) TC; (**B**) TGs; (**C**) GLU-YA; (**D**) ALT; (**E**) AST; (**F**) LDL-C; (**G**) HDL-C. Results are presented as means and SEMs; n = 6; * *p* < 0.05, ** *p* < 0.01.

Figure 3 demonstrates the impact of hawthorn polysaccharide supplementation on the serum lipid levels of mice kept on a high-fat diet. In contrast to the NC group, the high-fat diet significantly increased the serum levels of TC (4.61 ± 0.50 mmol/L, *p* < 0.05), ALT (27.13 ± 4.69 U/L, *p* < 0.05), and LDL-C (0.94 ± 0.12 mmol/L, *p* < 0.01) in the HC group mice. In contrast to the NC group, the levels of TC, ALT, and LDL-C of the HC group mice increased by 18.15%, 20.25%, and 32.85%, respectively. After supplementation with hawthorn polysaccharides, this trend was reversed. The TC, ALT, and LDL-C levels in the HA group mice were 4.38 ± 0.40 mmol/L, 20.17 ± 2.80 U/L (*p* < 0.01), and 0.75 ± 0.09 mmol/L (*p* < 0.05), respectively, which were significantly lower than those of the HC group. For the TG, AST, and GLU-YA indicators, non-significant differences were observed among the three groups of mice. Meanwhile, the serum HDL-C levels of the HC group mice decreased because of the high-fat diet. After hawthorn polysaccharide supplementation, the HDL-C level of the HA group mice increased to 4.60 ± 0.27 mmol/L, which was dramatically higher (by 17.85%) (*p* < 0.05) than in the HC group. HDL-C can remove cholesterol from the innermost walls of blood vessels, thereby reducing the risk of heart disease. In conclusion, hawthorn polysaccharides improved serum lipid levels in mice on a high-fat diet, leading to a healthier state.

### 3.5. Liver Antioxidant Indicators

**Figure 4:**
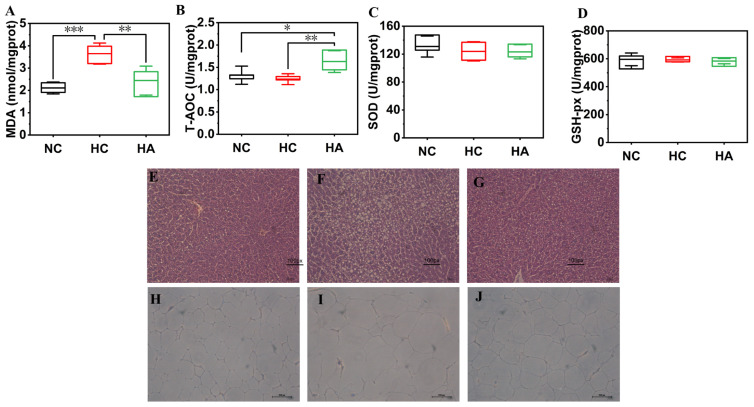
Liver Antioxidant Indicators for (**A**) MDA; (**B**) T-AOC; (**C**) SOD; (**D**) GSH-px. Results are presented as means and SEMs; n = 6; * *p* < 0.05, ** *p* < 0.01, *** *p* < 0.001. Images of H&E staining for liver in 200× magnification for (**E**) NC; (**F**) HC; and (**G**) HA, and epididymal fat in 200× magnification for (**H**) NC; (**I**) HC; and (**J**) HA.

Abnormal lipid metabolism generates a large number of free radicals, thereby triggering oxidative stress responses. By measuring the antioxidant-related indicators in mouse livers (Figure 4), it was found that, in contrast to the NC group, the activity of T-AOC in mice of the HC group was significantly reduced (1.24 ± 0.12 U/mgprot, *p* < 0.05), while the MDA level was significantly increased (3.65 ± 0.47 nmol/mgprot, *p* < 0.001), demonstrating that the antioxidant defense system of the high-fat blood disease mice was damaged and obvious oxidative stress damage occurred [33,34,35]. After the oral administration of hawthorn polysaccharides, these indicators were effectively improved. In contrast to the HC group, the T-AOC activity increased by 31.92% (*p* < 0.01), and the MDA level was reduced significantly by more than 33.12% (*p* < 0.01) in the HA group. The research findings suggest that hawthorn polysaccharides could effectively exclude excessive free radicals, reduce lipid peroxide generation, enhance antioxidant capacity, and alleviate the damage caused by oxidative stress.

### 3.6. Histological Analysis

H&E staining was executed on the liver of every mouse to observe the liver damage in the hyperlipidemic mice. Figure 4E–G illustrate the microscopic morphological characteristics of hepatic cells. Hepatocytes in the NC group displayed a well-preserved cellular structure, arranged regularly, with clear cell boundaries, and only a few small fat particles were present. There was no infiltration of inflammatory cells, and no apparent liver damage was observed. Following an 8-week high-fat-fed intervention, hepatocytes in the HC group exhibited severe steatosis with prominent lipid vacuole formation and disorganized cellular arrangement. The liver cell structure was significantly damaged. The addition of hawthorn polysaccharides reduced liver injury attributed to the high-fat feeding, and liver tissue morphology was significantly restored, approaching that of mice in the NC group. The quantity and size of the fat vacuoles of the liver cells in the HA group were observably lessened, and the liver cell structure was significantly improved. Collectively, these results demonstrate that hawthorn polysaccharides can effectively alleviate hepatic cell injury and fat accumulation caused by a high-fat diet.

Histopathological observation of epididymal fat was conducted through H&E staining. The results revealed significant variations in both the size and quantity of adipocytes across the different treatment groups (Figure 4H–J). The adipocytes in the NC group mice were regular in size and distribution, while those in the HC group had significantly larger volumes and a more disordered arrangement. The same results were also found in the research of [36]. Although the volume of adipocytes in the hawthorn polysaccharide treatment group (HA group) was still larger than that in the NC group, it was memorably lessened in contrast to the HC group, suggesting that hawthorn polysaccharides can effectively inhibit fat accumulation following high-fat diet administration.

### 3.7. The Impact on the Intestinal Flora

**Figure 5:**
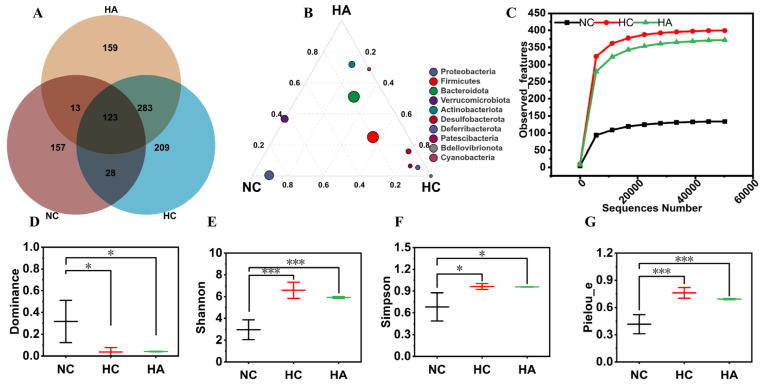
The effects of hawthorn polysaccharide supplementation on the richness and α-diversity of the intestinal microbial structure in the high-fat-diet mice. (**A**) The Venn diagram of the operational classification units (OTUs) of the three groups. (**B**) The ternary diagram explains the differences in the relative abundances of various types of microorganisms among the NC, HC, and HA groups. (**C**) The microbial density curves of the NC, HC, and HA groups. (**D**) Dominance index. (**E**) Shannon index. (**F**) Simpson index. (**G**) Pielou_e index. Data presented as mean SEM; n = 6; * *p* < 0.05, *** *p* < 0.001.

Figure 5A displays the distribution of operational taxonomic units (OTUs) in groups, applying the Venn diagram, and provides a comparative analysis of common and unique OTUs across groups. In this study, the NC group had 321 OTUs, the HC group had 577 OTUs, and the HA group had 643 OTUs. This indicates that, among the groups, the HA group had the maximum species richness. There were 123 common OTUs among the three groups, while the unique OTUs specific to the NC, HC, and HA groups were 157, 159, and 209, respectively. The differences in the number of OTUs among the three groups indicate significant differences in the intestinal microbiota structure of the mice. The ternary plot in Figure 5B shows differences in gut microbiota composition at the phylum level among the three groups. As shown in Figure 5B, each point in the ternary plot corresponds to a specific microbial community category. The results indicated that, except for *Proteobacteria* in the NC group and *Bdellovibrionota* in the HC group, most of the dominant phyla clustered at the center of the triangular plot. This indicates that the communities under different dietary conditions are similar. Figure 5C shows the rarefaction curves. It can be seen that the rarefaction curves of the NC, HC, and HA samples all gradually become gentler, indicating that the sequencing depths of each sample meet the requirements and are reasonable. The current sequencing volume can cover most of the species in this test.

Through Alpha diversity analysis, the differences in species munificence and microbial diversity in each mouse could be evaluated [30]. In this study, the Dominance, Shannon, Simpson, and Pielou_e indices were analyzed for the three groups. The Dominance index reveals the richness of the community. As depicted in Figure 5D, in contrast to the NC group, the HC and HA groups showed a pronounced reduction in the Dominance index (*p* < 0.05). On the contrary, the Shannon (*p* < 0.001), Simpson (*p* < 0.05), and Pielou_e (*p* < 0.001) indices of the HC and HA groups increased significantly. These research findings suggested that a high-fat diet can alter the richness and diversity of the microbial community. Interestingly, compared with the high-fat-diet group, the addition of hawthorn polysaccharides did not change these richness and diversity indicators (*p* > 0.05).

**Figure 6:**
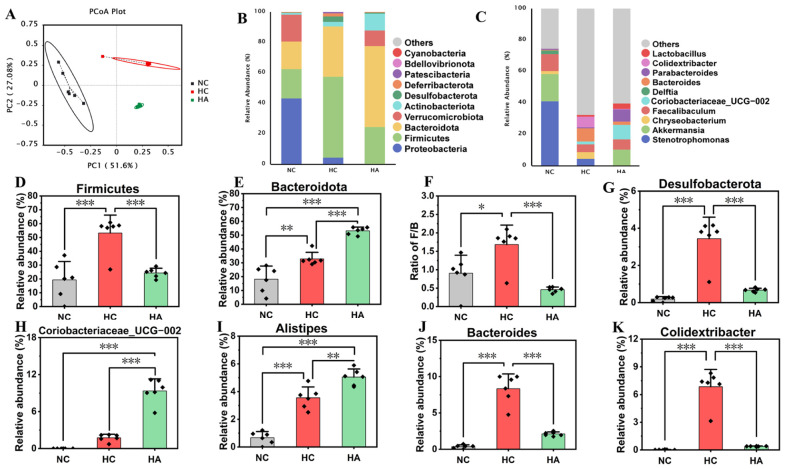
The impact of hawthorn polysaccharides on the intestinal flora structure in mice fed a high-fat diet. (**A**) The results of principal coordinate analysis (PCoA) of the gut microbiota in the NC, HC, and HA groups. (**B**) The compositional profiles of gut microbiota at the phylum level. (**C**) The compositional proportion of gut microbiota at the genus level. (**D**) Relative abundance of *Firmicutes*. (**E**) Relative abundance of *Bacteroidota*. (**F**) Ratio of F/B. (**G**) Relative abundance of *Desulfobacterota*. (**H**) Abundance of *Coriobacteriaceae*_UCG-002. (**I**) Relative abundance of *Alistipes*. (**J**) Relative abundance of *Bacteroides*. (**K**) Relative abundance of *Colidextribacter*. Data presented as mean SEM; n = 6; * *p* < 0.05, ** *p* < 0.01, *** *p* < 0.001.

At the phylum level, by analyzing the relative abundances of major taxa (the top 10 by mean abundance), it was found that the dominant taxa and the Firmicutes/Bacteroidota ratio (ratio of F/B) in the gut microbiota of the three groups of mice changed due to different diets (Figure 6B,D–G). Proteobacteria, Firmicutes, and Bacteroidota were the predominant bacterial species in each group. In the HA group, Proteobacteria disappeared. The HC group presented a relative abundance of Firmicutes of 52.23% ± 12.98%, which was markedly greater than that in the NC group (19.32% ± 13.27%, *p* < 0.001). Regarding the relative abundance of Bacteroidota, in the HC group, the relative abundance was 32.83% ± 4.76%, notably higher than that in the NC group (18.17% ± 9.45%, *p* < 0.01). In contrast to the HC group, the intervention of HA observably increased the abundance of *Bacteroidota* (53.18% ± 2.66%, *p* < 0.001), which was 62.01% higher than that in the HC group, but had a significantly inhibitory impact on the relative abundance of Firmicutes (24.32% ± 3.39%, *p* < 0.001), which was 54.31% lower than that in the HC group. The results of the changes in the relative abundances of Firmicutes and Bacteroidota showed that, in the HC group, F/B exhibited a pronounced increase (*p* < 0.05). After the supplementation of hawthorn polysaccharides, the F/B ratio showed a noteworthy decline (*p* < 0.001) in the HA group. These findings indicate that the supplementation of hawthorn polysaccharides can improve the intestinal microbiota structure related to obesity, which agrees with the research of Yue S.-J. et al. (2023) [37]. The high-fat diet induced a significant augmentation in the relative abundance of *Desulfobacterota* [37] in contrast to the NC group (*p* < 0.001) [37]. After the addition of hawthorn polysaccharides, the relative abundance of *Desulfobacterota* noteworthily decreased (*p* < 0.001), bringing its level closer to that of the NC group.

As for genus (as depicted in Figure 6C), both high-fat feeding and hawthorn polysaccharide supplementation significantly modified the relative abundance of dominant intestinal flora. In contrast to the standard diet, a high-fat diet led to a remarkable rise in the relative abundance of *Coriobacteriaceae* UCG-002 (Figure 6H), Alistipes (Figure 6I), and *Colidextribacter* (Figure 6K). After hawthorn polysaccharide supplementation, it inhibited the increase in the relative abundances of *Colidextribacter* induced by the high-fat diet. The relative abundances of *Colidextribacter* in the HA group were significantly lower than those in the HC group (Figure 6K, *p* < 0.001). At the same time, the relative abundances of Lactobacillus in the HA group were markedly increased (Figure 6J, *p* < 0.01). Lactobacillus is commonly found in the intestines and fermented foods; some strains express bile salt hydrolase (BSH), which deconjugates bound bile acids to generate free bile acids, and participate in cholesterol metabolism. This indicates that the hawthorn polysaccharides increase the relative abundance of flora related to bile salt metabolism, thereby improving hyperlipidemia.

### 3.8. LEfSe Algorithm Analysis

**Figure 7:**
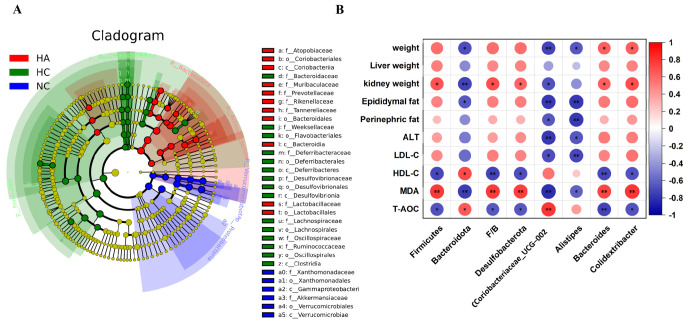
The LEfSe analysis of the gut microbes of mice. (**A**) Cladogram with an LDA score > 4.0. (**B**) Correlation between the abundance of different species and the indicators of hyperlipidemia. * *p* < 0.05, ** *p* < 0.01.

LEfSe analysis is a means for identifying key species that exhibit remarkable differences in relative abundance between different groups. The selected species that showed significant differences could be identified as the key microbial markers for distinguishing between the groups. As given in Figure 7A,B, the predominant bacterial taxa in the NC group are c_*Gammaproteobacteria*, p_*Proteobacteria*, o_*Xanthomonadales*, f_*Xanthomonadaceae*, and g_*Stenotrophomonas*. The high-fat diet alters the dominant bacterial species. The HC group was identified by c_*Clostridia*, p_*Firmicutes*, o_*Oscillospirales*, and f_*Oscillospiraceae*. However, the HA group had a high abundance of o_*Bacteroidales*, p_*Bacteroidota*, and c_*Bacteroidia* f_*Muribaculaceae*. The above-mentioned bacterial groups may play an essential role in adjusting the intestinal microbial structure through hawthorn polysaccharides.

### 3.9. Correlation Between Intestinal Flora and the Hyperlipidemia-Related Indices

To further analyze the association between the relative abundances of different species and hyperlipidemia indicators, a correlation analysis was conducted on the data of these two parts. The results are presented in Figure 7B. In particular, at the phylum level, Firmicutes, F/B, and *Desulfobacterota* were significantly positively correlated with MDA (r = 0.77, r = 0.76, and r = 0.74, respectively) and kidney weight (r = 0.67, r = 0.68, and r = 0.61, respectively). *Firmicutes*, F/B, and *Desulfobacterota* were meaningfully inversely correlated with HDL-C (r = −0.71, r = −0.73, r = −0.69, respectively). At the genus level, Coriobacteriaceae_UCG-002 was significantly positively related to T-AOC (r = 0.78) and significantly negatively correlated with MDA (r = −0.81), ALT (r = −0.77), epididymal fat (r = −0.77), and weight (r = −0.72). At the same time, Alistipes was significantly and negatively correlated with LDL-C (r = −0.71), epididymal fat (r = −0.77), perinephric fat (r = −0.77), and weight (r = −0.64). Additionally, both Bacteroides and Colidextribacter were significantly negatively correlated with T-AOC (r = −0.72, r = −0.67, respectively) and HDL-C (r = −0.72, r = −0.67, respectively), but significantly positively correlated with MDA (r = −0.73, r = 0.78, respectively), kidney weight (r = −0.63, r = −0.68, respectively), and weight (r = −0.63, r = 0.64, respectively).

## 4. Discussion

Hyperlipidemia is a metabolic disorder characterized by dysregulated fatty substance metabolism, which poses a risk to people’s health worldwide. It is often accompanied by weight gain and obesity [38]. Polysaccharides are bioactive compounds widely present in plant-based foods. Dietary polysaccharides are considered safe and exhibit strong antioxidant, antiviral, and antitumor activities [38]. Dietary supplementation of polysaccharides can also lower blood lipids by reducing low-density lipoprotein and cholesterol levels, such as polysaccharides from Rice Bran [7] and Astragalus [37], which all improve disorders of lipid metabolism. Therefore, based on current reports from hawthorn research [21,22,25,26], it is necessary to explore whether hawthorn polysaccharides improve carbohydrate and lipid metabolism imbalances in vivo.

In this study, hawthorn polysaccharides could mitigate high-fat-diet-induced hyperlipidemia. In contrast to the HC group, the administration of HA markedly reduced body weight (*p* < 0.001). The body weight of the HA group mice was approximately the normal level of the NC group (*p* > 0.05). In addition, compared to the HC group, hawthorn polysaccharide addition also noteworthily lessened the weights of organs (liver, *p* < 0.05; kidney, *p* < 0.01), perirenal fat (*p* < 0.05), and epididymal fat (*p* < 0.01) in the HA group. Especially, the index of epididymal fat (*p* < 0.01) was reduced. This indicates that a hawthorn polysaccharide intervention can reduce fat accumulation. Therefore, hawthorn polysaccharides alleviate hyperlipidemia by decreasing the stacking of fat in the body [39]. Similarly, Yang et al. (2020) found that a flaxseed polysaccharide intervention significantly reduced epididymal adipose tissue weight in mice receiving high-fat feeding [15]. The continuous high-fat diet led to significantly higher TC and ALT levels in the HC group than in the NC group (*p* < 0.05). After the intervention with hawthorn polysaccharides, the TC level in the HA group was reduced, although there was no statistically significant difference from the HC group (*p* > 0.05). Studies have shown that an increase in TC levels may indicate an increased risk of cardiovascular diseases [39]. The results of this study indicate that the hawthorn polysaccharide intervention can lower TC levels and reduce the risk of vascular disease. The same results were obtained in the study of Liu et al. (2024) [40]. Meanwhile, in contrast to the NC and HC groups, the HA group displayed significantly higher HDL-C (*p* < 0.05) and lower LDL-C (*p* < 0.05). This finding is in accordance with the research by Ge et al. [41]. Studies have shown that LDL-C can transport TC to peripheral tissues, leading to abnormal lesions in the vascular walls [42]. In contrast, HDL-C can transport TC to the liver and may provide protective effects against various vascular diseases [43]. The effects of plant-derived natural polysaccharides in improving hyperlipidemia have been widely studied, and various mechanisms of lipid-lowering action have been investigated. For instance, Yang et al. studied the lipid-lowering impact of *Cyclocarya paliurus* polysaccharides. The results indicated that the polysaccharides from *Cyclocarya paliurus* could ameliorate hyperlipidemia at the molecular level by modulating the expression of genes associated with fatty acid synthase and acetyl-CoA carboxylase [33]. Meanwhile, according to the research by Hu et al. [44], the polysaccharides of *Cyclocarya paliurus* can modulate the activities of fat metabolism-related enzymes, thereby ameliorating hyperlipidemia.

Furthermore, the hawthorn polysaccharides reduced the ALT levels in the HA group (*p* < 0.01) to the same as those in the NC group (*p* > 0.05). Zou et al. (2023) investigated the effects of polysaccharide-rich fractions from Enteromorpha prolifera on lipid metabolism abnormalities induced by a high-fat diet, and obtained the same results [45]. Studies have shown that even a small amount of liver cell damage will cause a significant increase in ALT levels. The ALT level can be used to assess the degree of liver damage [46]. Therefore, this result indicates that the high-fat diet induced oxidative stress in the livers of mice in the HC group. The main manifestation is that the MDA level was extremely significantly increased (*p* < 0.001) compared to the NC group. The present result aligns with the observations of Peng et al. (2024) [47]. Meanwhile, in the liver, SOD and GSH are key enzymes that eliminate excess ROS and maintain redox balance [48]. This study measured the enzyme activities in the liver. The findings suggested that, compared to the NC group, the levels of T-AOC and the SOD and GSH activities in the high-fat-diet mouse liver were all decreased. However, after the intervention with hawthorn polysaccharides, the HA group mice exhibited a notable reduction in MDA levels (*p* < 0.01), suggesting that hawthorn polysaccharides have a certain effect in improving the oxidative stress-induced injury in the mice’s livers. The intervention with hawthorn polysaccharides can effectively alleviate liver damage caused by a high-fat diet. Furthermore, in this study, through the pathological liver sections of mice fed a high-fat diet, it could be observed that hepatic cells exhibited a disordered arrangement and were accompanied by cellular damage. The number of fat vacuoles increased, with significant fat accumulation, which agreed with the research of [29]. Hawthorn polysaccharides can reverse this damage. After 8 weeks of intragastric administration, the liver cell morphology in the mice improved significantly, and liver fat accumulation was significantly reduced. This result is consistent with the results of Liu et al. (2024) [40] and Li et al. (2023) [20]. The above results indicate that hawthorn polysaccharides can alleviate liver damage and abnormal fat accumulation induced by a high-fat diet. The intestinal microbiota is closely linked to lipid metabolism. The complex intestinal microbiota system affects various diseases. Therefore, maintaining the steady state of the intestinal microbiota’s structure is important [49]. Previous research has shown that some plant polysaccharides can reduce blood lipids through improving intestinal microbiota structure [50,51]. Therefore, in the present study, 16S rRNA sequencing technology was used to detect the microflora in the feces of mice. The results showed that a long-term high-fat diet reduced intestinal microbial richness and diversity in mice in the HC group and increased the F/B ratio. The increase in the F/B ratio indicates an augmentation in the bacterial community associated with obesity and a decrease in the bacterial community that resists obesity [52]. Furthermore, the decrease in the abundance of *Firmicutes* is relevant to increased consumption of secondary bile acids. Disruption of the intestinal flora can destroy the normal balance of bile acid metabolism, leading to additional production of secondary bile acids, which in turn cause liver injury and exacerbate inflammation, promoting the occurrence or development of non-alcoholic fatty liver disease. This highlights the importance of the microbiota-related bile acid profile for metabolic health.

The research results suggested that the *Bacteroidota* phylum is inversely related to obesity. Similar to this result, Wang et al. noted that, in the high-fat-diet-fed mice, the F/B ratio significantly decreased after an intervention with yushu polysaccharides [53], which is consistent with previous research [54,55]. This research found that, in contrast to the normal control (NC) mice, the relative abundances of *Desulfobacterota* and *Colidextribacter* of mice in the HC group were markedly elevated (*p* < 0.001). The research indicates that *Desulfobacterota* and *Colidextribacter* may be important bacterial groups contributing to obesity [55]. After the addition of hawthorn polysaccharides, the relative abundance of *Bacteroidota*, which is inversely correlated with obesity (*p* < 0.001), was markedly decreased, as was the F/B ratio (*p* < 0.001). Additionally, the richness of *Desulfobacterota* and *Colidextribacter* in the HA group was observably reduced (*p* < 0.001). Therefore, the effect of hawthorn polysaccharides in preventing obesity may be related to the elevated proliferation of salutary bacterial taxa, including Bacteroidota, and the diminished proliferation of unhealthy taxa, for instance, *Firmicutes*. Furthermore, the intervention with hawthorn polysaccharides also significantly increased the proliferation of *Coriobacteriaceae* UCG-002. The findings suggest that *Coriobacteriaceae* UCG-002 is highly correlated with changes in the bile acid profile. This indicates that hawthorn may influence bile acid metabolism by regulating these specific bacterial communities. This discovery provides a new perspective for understanding the potential effect of hawthorn polysaccharides on the enteric canal microbiota and host metabolism. In conclusion, the changes in the types and quantities of the microbial communities in the intestinal tract, as well as the alterations in species richness and diversity caused by an HFD, can all be reversed by Schisandra polysaccharides. Liu et al. [56] also found that astragalus polysaccharides alleviate non-alcoholic fatty liver disease by increasing the quantity of helpful bacteria and diminishing that of morbific bacteria. In conclusion, hawthorn polysaccharides are primarily fermented by colonic microorganisms, thereby adjusting the intestinal flora and improving fat metabolism. The core mechanisms are regulating the gut microbiota, increasing the quantity of helpful bacteria (*Lactobacillus*, *Bacteroidota*), decreasing potential unhealthy bacteria (*Desulfobacterota*, *Colidextribacter*), promoting the production of short-chain fatty acids (SCFAs), increasing HDL-C levels, decreasing LDL-C levels, improving cholesterol metabolism, improving liver lipid metabolism, and ultimately reducing fat deposition. The above results indicate that hawthorn polysaccharides have excellent anti-obesity and lipid-lowering effects and significantly regulate specific bacterial genera, thereby exerting a protective effect in lowering blood lipids.

Research has not yet explored the impact of a hawthorn polysaccharide intervention on short-chain fatty acids, which merits further in-depth study. In future studies, attention can be paid to the impact of plant polysaccharides on gut microbiota metabolites to provide a more detailed explanation of the theory that plant polysaccharides reduce blood lipids.

## 5. Conclusions

This experiment used high-lipid model mice as experimental subjects. After 8 weeks of the intragastric administration of hawthorn polysaccharides, the findings showed that the body weight of the HA group mice was markedly lower than that of the HC group and close to that of the NC group. In addition, the organ weights of mice in the HC group were dramatically enhanced; fat accumulation was evident, and serum indicators showed elevated lipid levels, with TC levels increasing significantly. The hawthorn polysaccharide intervention improved these symptoms. In the HA group, liver and kidney weights in mice were significantly reduced in contrast to the HC group; fat mass was also observably reduced, and the TC level was markedly lower than that in the mice in the HC group. Moreover, the addition of hawthorn polysaccharides significantly increased HDL-C and decreased LDL-C in the HA group mice. Furthermore, gut microbiota analysis demonstrated that high-fat feeding altered its structure. The intervention with hawthorn polysaccharides could modulate the intestinal flora, decrease the F/B ratio, and increase the abundance of beneficial bacterial strains, which is associated with lower blood lipid levels. In summary, this study demonstrates that hawthorn polysaccharides can alleviate high blood lipid symptoms caused by high-fat feeding. This is possibly attributed to its effects on lipid metabolism, including alleviating liver injury and abnormal fat accumulation, inhibiting oxidative stress, and regulating the structure of the enteric microorganisms. The findings of this study lay a theoretical foundation for applying hawthorn polysaccharides in lipid-lowering foods or functional foods.

## Data Availability

The original contributions presented in this study are included in the article. Further inquiries can be directed to the corresponding author.
